# Optical Coherence Tomography of Retinal and Choroidal Tumors

**DOI:** 10.1155/2012/385058

**Published:** 2011-06-08

**Authors:** Emil Anthony T. Say, Sanket U. Shah, Sandor Ferenczy, Carol L. Shields

**Affiliations:** Oncology Service, Wills Eye Institute, Thomas Jefferson University, Suite 1440, 840 Walnut Street, Philadelphia, PA 19107, USA

## Abstract

Optical coherence tomography (OCT) has revolutionized the field of ophthalmology since its introduction 20 years ago. Originally intended primarily for retina specialists to image the macula, it has found its role in other subspecialties that include glaucoma, cornea, and ocular oncology. In ocular oncology, OCT provides axial resolution to approximately 7 microns with cross-sectional images of the retina, delivering valuable information on the effects of intraocular tumors on the retinal architecture. Some effects include retinal edema, subretinal fluid, retinal atrophy, photoreceptor loss, outer retinal thinning, and retinal pigment epithelial detachment. With more advanced technology, OCT now provides imaging deeper into the choroid using a technique called enhanced depth imaging. This allows characterization of the thickness and reflective quality of small (<3 mm thick) choroidal lesions including choroidal nevus and melanoma. Future improvements in image resolution and depth will allow better understanding of the mechanisms of visual loss, tumor growth, and tumor management.

## 1. Introduction

Since its inception in 1991, optical coherence tomography (OCT) has found wide applications in medicine including gastroenterology, dermatology, cardiology, and ophthalmology [1–4]. Traditional time domain OCT, sold commercially in 1995 and used primarily by retina and glaucoma specialists, has been largely replaced by Spectral or Fourier domain OCT that provides higher resolution images (4–7 um) and faster scanning speeds (up to 40,000 scans per second) that could translate to broader application of OCT for other ophthalmic subspecialties including pediatric ophthalmology, oculoplastics, and ocular oncology [5–8]. 

OCT is a valuable diagnostic tool for evaluation of tissue architecture of the postequatorial fundus (inner retina, outer retina, retinal pigment epithelium (RPE), and choroid). In ocular oncology, OCT allows for diagnosis, treatment planning, and monitoring response. Traditionally, OCT was primarily used to image the neurosensory retina and the retinal pigment epithelium (RPE) with outstanding resolution, but the choroid and sclera have been poorly imaged. Today, software upgrades and new imaging techniques allow longer scan lengths, enhanced depth imaging (EDI), and three-dimensional reconstruction. These newer features allow demonstration of more peripheral tumors, higher resolution images of anatomy deep to the retina, and improved characterization of intraocular tumors [8–10]. Herein we review clinical features of posterior segment intraocular tumors on OCT and its applications in the management of these lesions. 

## 2. Choroidal Nevus

Choroidal nevi are the most common intraocular tumor. Population studies show higher prevalence of these tumors in Caucasians (6.5%) compared to Asians (1.4%) [11]. Nevi are typically pigmented, with smooth margins and with overlying drusen, measuring less than 5 mm in basal diameter and 3 mm in thickness. They often do not cause visual symptoms and more importantly are generally benign. It has been estimated, however, that 1 in 8845 choroidal nevi undergoes malignant transformation into melanoma [12]. Although the odds appear minimal, careful evaluation and followup of all choroidal nevi is advised. Factors predictive of nevus transformation into melanoma include thickness greater than 2 mm, the presence of subretinal fluid, orange pigment, juxtapapillary location, and symptoms of blurred vision or photopsia [13]. The presence of any one factor gives a relative risk of 1.9, three factors 7.4, and the presence of all five will give a relative risk of 27.1 [14]. 

**Figure 1:**
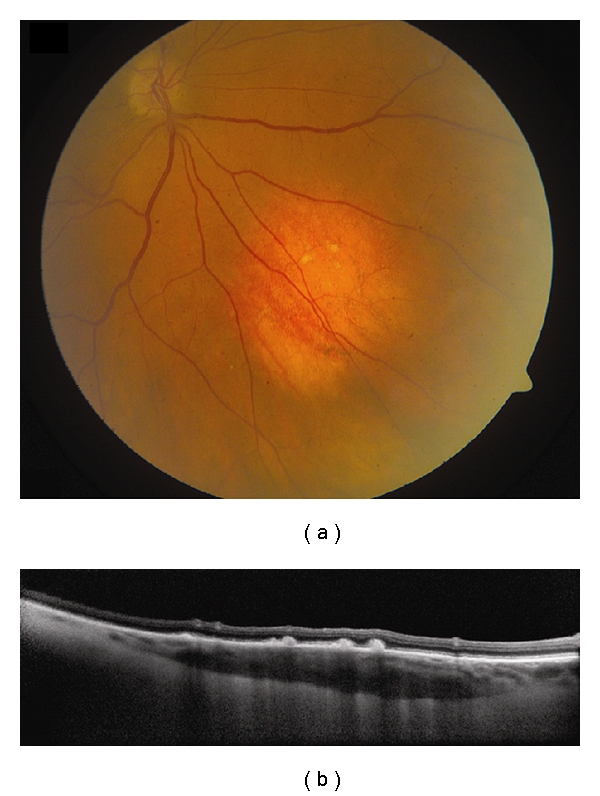
Choroidal nevus. (a) Amelanotic choroidal nevus with overlying RPE alterations and areas of RPE atrophy. (b) EDI OCT image shows both anterior and posterior margins of the lesion. There is gradual transition between the hyperreflective inner choroid and hyporeflective outer choroid. There is loss of choriocapillaris over the main lesion. Multifocal excrescences of the RPE are also present, suggestive of drusen.

OCT features of choroidal nevus have been extensively documented but are limited mostly to its effects on the overlying retina and the anterior choroidal surface [8]. Shields and associates compared the frequency of retinal findings by clinical examination to OCT [15]. They found that OCT has a higher sensitivity than clinical examination in detection of overlying retinal edema (15% by OCT versus 3% by clinical examination), subretinal fluid (26% versus 16%), retinal thinning (22% versus 0%), and RPE detachment (12% versus 2%) [15]. OCT also enabled the examiners to characterize retinal edema (cystoid versus noncystoid) and determine the status of overlying photoreceptors [15]. These features are significant, since foveal edema and RPE detachment were found to be predictive of 3 or more lines of vision loss (RR = 22.16 and 9.02, resp.) and a final visual outcome worse than 20/200 (RR = 12.80 and 18.72, resp.) [16]. Overlying photoreceptor loss can also explain associated visual field defects in some patients. Findings localized to the RPE are also visualized readily by OCT. OCT evidence of overlying drusen manifests as small dome-shaped elevations at the level of the RPE/Bruch's membrane [15] (Figure 1). Nevus-related drusen are found in 41% of choroidal nevi imaged by OCT and are also visualized by ophthalmoscopy [15]. 

On OCT, the choroidal findings in nevi are limited to the anterior surface and include hyporeflectivity in 62%, isoreflectivity in 29%, and hyperreflectivity in 9% [15]. Anterior choroidal reflectivity is affected by overlying RPE alterations and the amount of pigmentation [15]. The OCT findings reflect pigment within the mass and do not correlate to internal reflectivity and acoustic quality by ultrasonography, which imply density of cellularity [15]. 

## 3. Choroidal Melanoma

Uveal melanoma is the most common primary intraocular malignancy, and 90% develop in the choroid [17]. They often present as a pigmented, elevated, choroidal mass with associated orange pigment and subretinal fluid. Most choroidal melanomas can be differentiated from benign nevi because melanoma is much larger in size. However, approximately 30% of choroidal melanomas are small (≤3 mm thickness) and difficult to differentiate from nevi by clinical examination alone [17]. In these cases, OCT can be helpful in the detection of melanoma-related features in the overlying retina such as subretinal fluid [15]. Subretinal fluid associated with melanoma shift with positioning and may cause intermittent blurred vision or flashes. Overall, 15% and 25% of uveal melanomas metastasize in 5 and 10 years [17]. Shields and associates found subretinal fluid to be a significant risk for metastasis in 8033 cases of uveal melanoma, so detection of even subtle subretinal fluid by OCT could be vital to patient prognosis [17]. 

**Figure 2:**
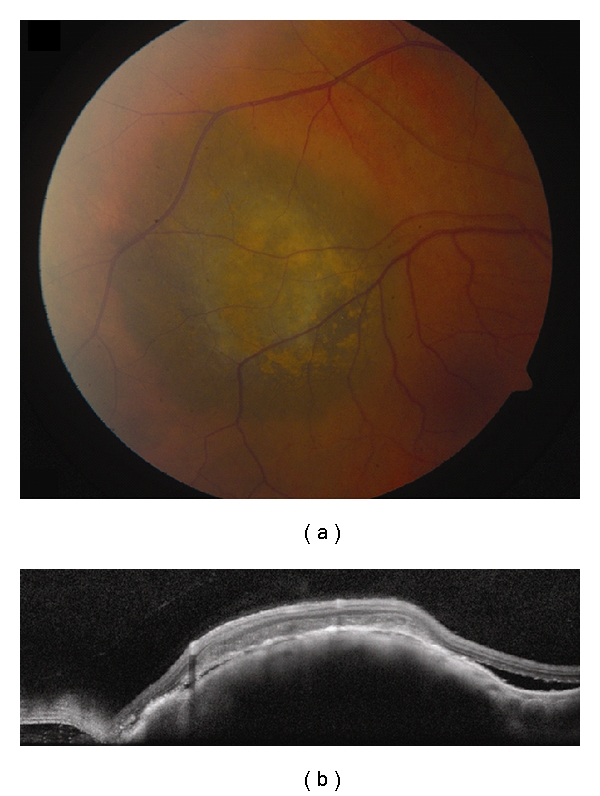
Choroidal melanoma. (a) Small choroidal melanoma with overlying RPE hyperplasia and diffuse orange pigment accumulation. (b) Spectral domain OCT clearly demonstrates subretinal fluid that could have been missed by clinical examination alone. Overlying the dome-shaped elevation of the choroid is a thickened irregular RPE and thickening of the outer retinal layers.

Subretinal fluid is an important characteristic related to underlying choroidal melanoma. Muscat and coworkers studied 20 untreated choroidal melanoma and detected subretinal fluid using time domain OCT in all cases [18]. Espinoza and colleagues also used time domain OCT to describe an active OCT pattern, wherein a localized serous retinal detachment was associated with an overlying retina of normal thickness, a feature that was highly associated with documented tumor growth (*P* = 0.033) and future treatment (*P* = 0.014) [19]. In contrast, a chronic OCT pattern, wherein the overlying retina was thinned, contains intraretinal cysts and with RPE thickening was associated with a long-standing lesion more likely to remain dormant [19]. Sayanagi and coworkers used 3D spectral domain OCT and found a significantly higher prevalence of subretinal fluid (91% versus 14%), retinal edema (61% versus 14%), and subretinal deposits (61% versus 11%) in choroidal melanoma compared with nevi [10]. Singh and associates used spectral domain OCT to describe dispersed accumulation of subretinal deposits corresponding to orange pigment over a small choroidal melanoma that had not been found with time domain OCT [20]. Spectral domain OCT was also capable of detecting early vitreous seeding as highly reflective 20–30 micron spheroidal bodies in the vitreous [21]. The limitation of OCT for choroidal melanoma lies in the difficulty of imaging the overlying retina for large melanomas and the inability to image past the anterior choroidal surface [19]. Reflectivity of the anterior choroid in melanoma is variable even with spectral domain OCT [20] (Figure 2). 

In addition to examination of the overlying retina and RPE, OCT has been used to monitor treatment response and complications following radiotherapy for choroidal melanoma. Horgan and associates performed pre- and postplaque radiotherapy OCT and found that the mean time to onset of radiation maculopathy was 12 months [22]. The authors also reported that 17% had macular edema by OCT at 6 months, 40% at 12 months, and 61% at 24 months [22]. In comparison, radiation maculopathy was detected by clinical examination alone 1% by 6 months, 12% at 12 months, and 29% at 24 months [22]. Further, OCT enabled the authors to classify macular edema into extrafoveolar noncystoid (grade 1), extrafoveolar cystoid (grade 2), foveolar noncystoid (grade 3), mild-moderate foveolar cystoid (grade 4), and severe foveolar cystoid (grade 5) [22]. This qualitative classification correlated with quantification of central foveolar thickness. In such cases, both OCT and visual acuity can be used to monitor treatment response following laser photocoagulation, intravitreal anti-VEGF, and intravitreal triamcinolone for radiation macular edema. 

## 4. Choroidal Metastasis

The choroid is the most common site of metastasis in the eye because of its vascularity. Patients usually present with painless blurring of vision, and 66% will have a prior history of systemic cancer, most commonly the breast in women and the lungs in men [23]. Among the 34% without a history of systemic cancer, the lung is the most common primary site after workup [23]. Clinically, choroidal metastasis appears as a solitary, nonpigmented choroidal mass with associated shifting subretinal fluid [23]. They can occasionally be associated with overlying RPE alterations and brown pigment accumulation corresponding to lipofuscin.

**Figure 3:**
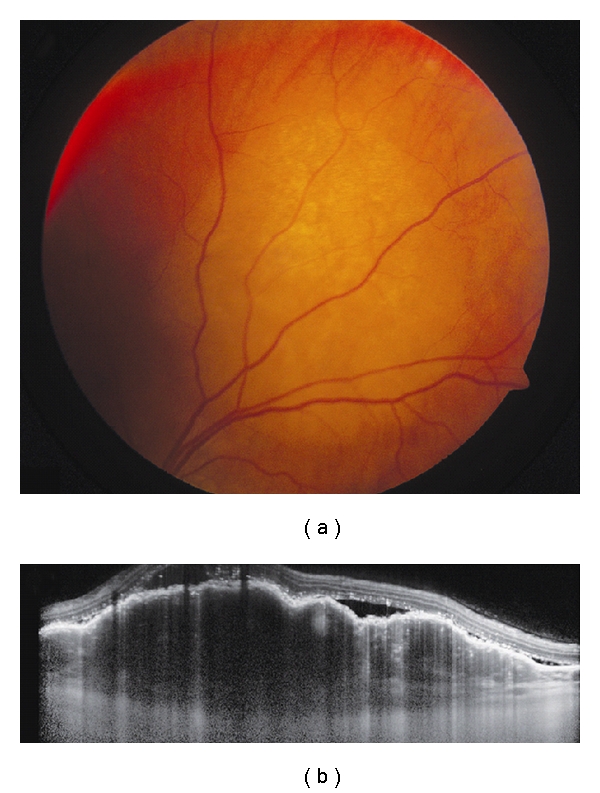
Choroidal metastasis. (a) Amelanotic choroidal metastasis in a patient with breast cancer. (b) EDI OCT reveals both the anterior and posterior margins of the metastasis allowing measurement of tumor thickness and characterization of its internal structure. Multiple nodular elevations of the RPE can also be seen with thickening of the RPE, overlying subretinal fluid, and hyperreflective deposits within the neurosensory detachments presumably from tumor cells or macrophages.

OCT of choroidal metastasis demonstrates a dome-shaped elevation of the neurosensory retina and RPE with adjacent subretinal fluid (Figure 3). It can also be associated with retinal edema, intraretinal cysts, and thickening and detachment of the RPE. Natesh and associates found highly reflective subretinal deposits corresponding to RPE clumping overlying the tumor on clinical examination [24]. Arevalo and colleagues also found highly reflective points within neurosensory detachment in 14.2% of cases and concluded that these points “may correspond to retinal compromise by cancer cells or macrophages containing lipofuscin and melanin granules” [25]. Choroidal features are also limited to the anterior surface as with all choroidal tumors, and they often have variable reflectivity [10]. In addition to its diagnostic capabilities, OCT is valuable in monitoring treatment response, since resolution of subretinal fluid and return of normal retinal architecture has been documented following therapy [25, 26]. 

## 5. Choroidal Hemangioma

Choroidal hemangiomas are benign vascular tumors that are either circumscribed or diffuse based on the extent of choroidal involvement [27]. Circumscribed choroidal hemangioma is usually orange colored, round, located in the posterior pole, and exhibits overlying retinal edema, subretinal fluid, and RPE alterations [28]. Choroidal hemangioma shows high internal reflectivity and acoustic solidity on ultrasonography, while often demonstrating a bright early filling and a characteristic late “wash out” on indocyanine green angiography [28]. On MRI, it is hyperintense to vitreous on T1, and isointense on T2, unlike most intraocular tumors, which are hypointense on T2 [28]. The diffuse variant usually extends to involve the entire choroid and is often associated with an ipsilateral facial hemangioma (nevus flammeus) that together comprise the Sturge-Weber syndrome [27, 28]. 

**Figure 4:**
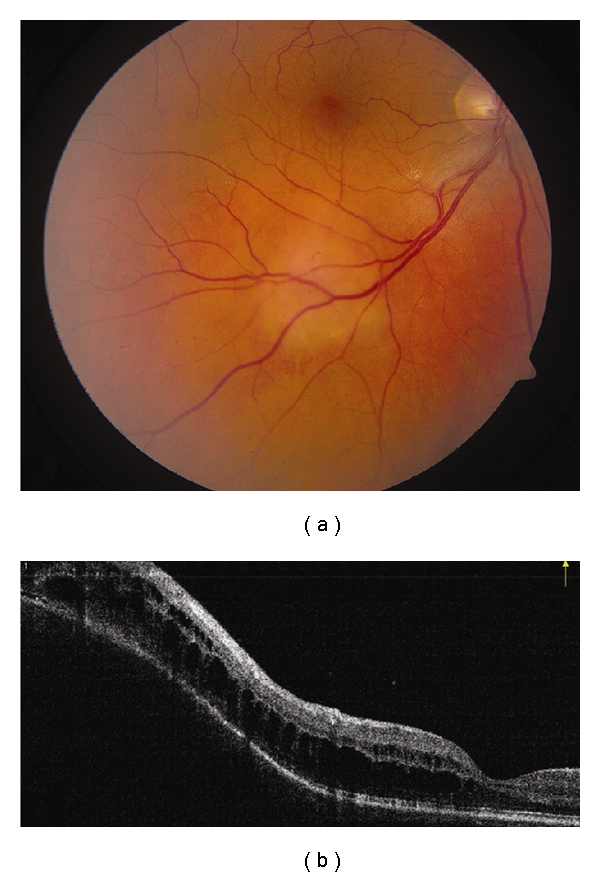
Choroidal hemangioma. (a) Choroidal hemangioma along inferotemporal arcade with subtle exudation seen inferonasal and inferotemporal to the foveola. (b) Time domain OCT shows dome-shaped elevation of its posterior border with overlying retinoschisis and retinal edema.

Ramasubramanian and colleagues described OCT findings in circumscribed choroidal hemangioma and found subretinal fluid (19%), retinal edema (42%), retinal schisis (12%), macular edema (24%), and localized photoreceptor loss (35%) [29]. The same group also described OCT findings in diffuse choroidal hemangioma and reported subretinal fluid (28%), retinal edema (14%), and photoreceptor loss (43%) [29]. Acute leakage from choroidal hemangioma demonstrates subretinal fluid with preserved photoreceptor layer and normal retinal thickness, whereas chronic leakage displays loss of photoreceptors, retinoschisis, and intraretinal edema associated with subretinal fluid [8] (Figure 4). By OCT, the anterior tumor surface is hyporeflective [10]. Currently, OCT has been used to monitor response to treatment by photodynamic therapy, transpupillary thermotherapy, plaque radiation, or laser photocoagulation. Blasi and associates performed photodynamic therapy on 25 cases of circumscribed choroidal hemangioma and reported a decrease in central foveal thickness and restoration of foveal anatomy following treatment after 5 years [30]. 

## 6. Choroidal Osteoma

Choroidal osteoma is a rare, osseous tumor often found in young females. This tumor appears as an orange-yellow plaque in the juxtapapillary region or macula but can demonstrate areas of whitening when decalcified. This tumor is benign but has the capacity to grow. Long-term studies have shown growth rates of 41–51%, choroidal neovascularization in 31–47%, and final visual outcome worse than 20/200 in 56–58% after 10 years [31, 32]. The mechanisms of visual loss include subretinal fluid, choroidal neovascularization, and photoreceptor loss. Shields and colleagues followed 74 eyes with choroidal osteoma to find subretinal fluid and tumor decalcification as factors predictive of poor visual outcome or loss of 3 or more lines of vision [32]. 

**Figure 5:**
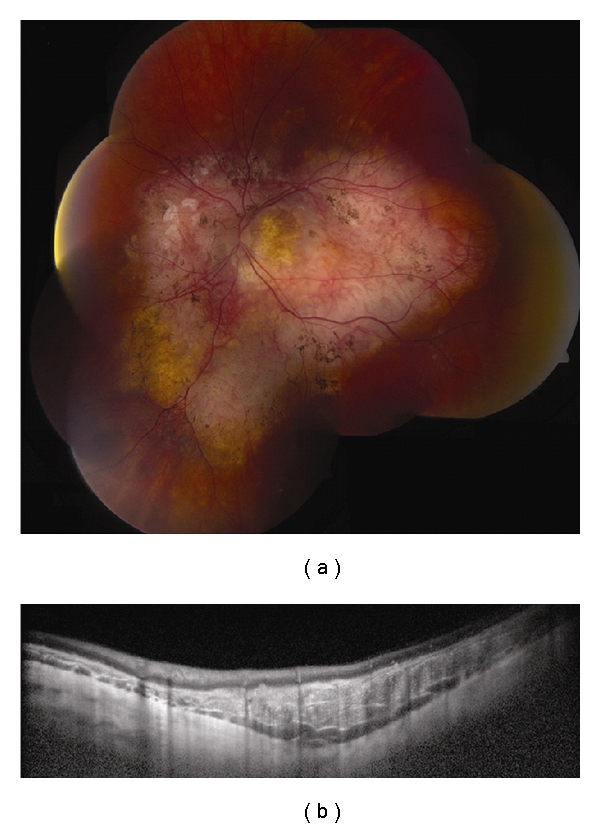
Choroidal osteoma. (a) Decalcified circumpapillary choroidal osteoma with associated pigment migration and RPE atrophy. (b) EDI OCT demonstrates replacement of the normal choriocapillaris with a dense hyperreflective mass with a scalloped posterior border and an adjacent hyporeflective space anterior to the sclera. The main lesion is almost continuous with the overlying RPE. Overlying neurosensory retina is thinned with the loss of the outer layers.

The internal structure of choroidal osteoma is difficult to evaluate with OCT and is limited to its anterior surface [8]. The overlying inner retina is often preserved while changes in the outer retinal layers, namely, the photoreceptors and the RPE, are often observed [8]. The RPE can sometimes be continuous with the inner surface of the underlying tumor, and the degree of calcification affects the amount of light transmission [8, 33, 34] (Figure 5). Shields and coworkers reported on the OCT features of choroidal osteoma and reported heterogeneity that largely depends on the amount of calcification [35]. Calcified portions of the tumor reveal mostly intact inner (100%) and outer (95%) retinal layers, a distinct RPE (57%), and mild transmission of light (95%) [35]. In contrast, decalcified portions of the tumor reveal intact inner retinal layers (90%), thinned outer retinal layers (100%), an indistinct RPE (90%), and marked light transmission into the tumor (70%) [35]. They also described focal areas of shadowing behind areas of RPE hyperplasia [35]. The anterior tumor surface was hyperreflective in 48% and isoreflective in 52% if calcified but was mostly hyperreflective (90%) when decalcified [35]. 

## 7. Lymphoid Tumors

Intraocular lymphoid tumors can occur in different parts of the eye with varying prognostic implications. There are two basic types, the vitreoretinal type and the uveal type. Vitreoretinal lymphoma accounts for most cases and are primarily diffuse large b-cell lymphomas [36]. They are aggressive tumors, highly associated with central nervous system lymphomas [36]. Patients are often elderly and immunocompetent or young and immunocompromised. Clinically, they present as bilateral multifocal yellowish deposits in the retina, subretina, or sub-RPE with overlying vitreous opacities. Pigment migration and RPE clumping can sometimes be visible overlying the tumor as brown leopard spots.

Uveal lymphoid tumors involve the choroid, ciliary body, and iris, often with conjunctival and orbital components. Most are extranodal marginal zone b-cell lymphoma although a benign reactive lymphoid hyperplasia subtype exists that presents similarly albeit less aggressive [36]. Choroidal lymphoid tumors are usually unilateral. They present as multifocal orange-yellow choroidal infiltrates resembling those from white dot syndromes [36]. In time, they can involve the entire uveal tract causing a diffuse thickening of the uvea on ultrasonography often with a small round echolucency behind the sclera [37]. The overlying retina and vitreous remain clear; however, the fornix or conjunctiva can be involved as “salmon patches” [36]. Compared to vitreoretinal lymphoma, uveal lymphomas are more indolent, but an association with systemic lymphoma exists.

**Figure 6:**
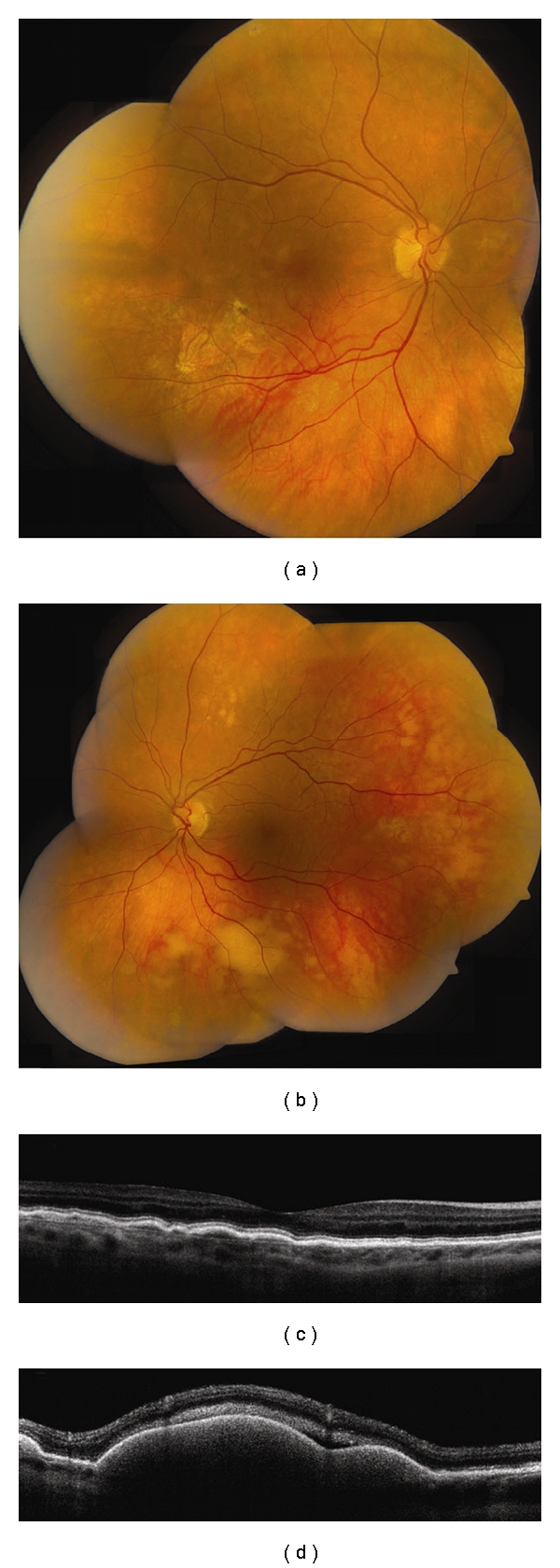
Vitreoretinal lymphoma. (a, b) Bilateral vitreoretinal lymphoma with multifocal cream-colored subretinal infiltrates. (c) Time domain OCT of the right eye reveals multiple nodular elevations of the RPE from deposits in the sub-RPE space. (d) Time domain OCT of the left eye shows multiple dome-shaped elevations of the RPE from more extensive infiltrates and subretinal fluid.

Diagnosis of all intraocular lymphoid tumors should be done with a biopsy, either from an associated conjunctival or forniceal involvement or through vitrectomy and fine needle biopsy of the involved ocular tissue. Ancillary testing is not absolutely necessary although it can provide insight on the extent of involvement. OCT in vitreoretinal lymphomas may show dome-shaped elevations of the RPE or small nodular RPE irregularities from sub-RPE tumor deposits, retinal elevation or thickening from tumor infiltration, and cystoid macular edema from associated inflammatory reaction [38–40] (Figure 6). Fardeau and associates examined 61 eyes with vitreoretinal lymphoma confirmed through vitreous biopsies and found that 41.7% had nodular elevations of the RPE [40]. The authors also reported a significantly thinner central foveal thickness compared to eyes with posterior uveitis other than lymphoma [40]. OCT performed in choroidal lymphoma shows regular intermittent placoid choroidal thickening and loss of choriocapillaris [41]. The overlying RPE and neurosensory retina is unaffected and retains its regular, smooth contour [41]. 

## 8. Congenital Hypertrophy of the Retinal Pigment Epithelium

Congenital hypertrophy of the retinal pigment epithelium (CHRPE) is a benign, flat, and pigmented lesion rarely associated with vision loss or visual field defects. This tumor is often unilateral and solitary but can occasionally be multifocal or grouped in a bear-track distribution. Familial adenomatous polyposis and Gardner's syndrome have been associated with a variant, wherein lesions appear in a haphazard multifocal distribution, and individual CHRPE has irregular borders and an atrophic “tail.” Shields and coworkers reported atrophic lacunae in 43% occupying a median 5% of the total area [42]. In their series of 330 patients with 337 lesions, growth in basal dimensions was documented by photographic comparison in 46%, while 5 (1.4%) lesions had a nodular elevation within CHRPE [42]. 

**Figure 7:**
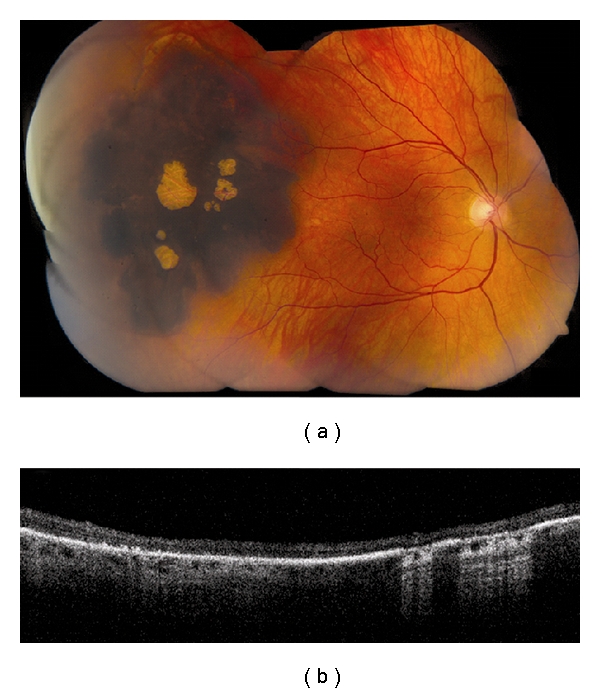
Congenital hypertrophy of the retinal pigment epithelium (CHRPE). (a) Large, peripheral CHRPE with centrally located lacunae. (b) Time domain OCT shows thinning of the overlying retina and loss of photoreceptors, posterior shadowing of the choroid in pigmented regions and light transmission to the underlying choroid in scans along lacunae.

OCT imaging of CHRPE can be difficult due to its peripheral location but could potentially be better captured using longer scan lengths by new-generation spectral domain OCT. Shields and colleagues described overlying retinal thinning and photoreceptor loss in all patients with CHRPE that likely account for visual field defects [43]. The neurosensory retina overlying CHRPE was only 68% the thickness of adjacent normal retina [43]. Pigmented CHRPE has 52% thicker RPE than adjacent normal retina that prevents light transmission and shadows the underlying choroid [43]. Nonpigmented CHRPE, on the other hand, has large areas of lacunae with thinner RPE that allow transmission of light and partial visualization of the choroid [43] (Figure 7). 

## 9. Combined Hamartoma of the Retina and Retinal Pigment Epithelium

A typical appearance of combined hamartoma of the retina and RPE is an elevated grey mass of the retina blending imperceptibly with surrounding retina and RPE without retinal detachment, or vitreous inflammation. There is often an associated preretinal fibrosis with traction on the adjacent retina. In a classic report by Schachat and associates from the Macula Society, vascular tortuosity was present in 93%, vitreoretinal surface abnormalities in 78%, pigmentation in 87%, and associated lipid exudation in 7% [44]. Shields and coworkers analyzed 77 cases based on macular versus extramacular location and reported more visual acuity loss ≥3 Snellen lines in the macular group (60% versus 13%) [45].

**Figure 8:**
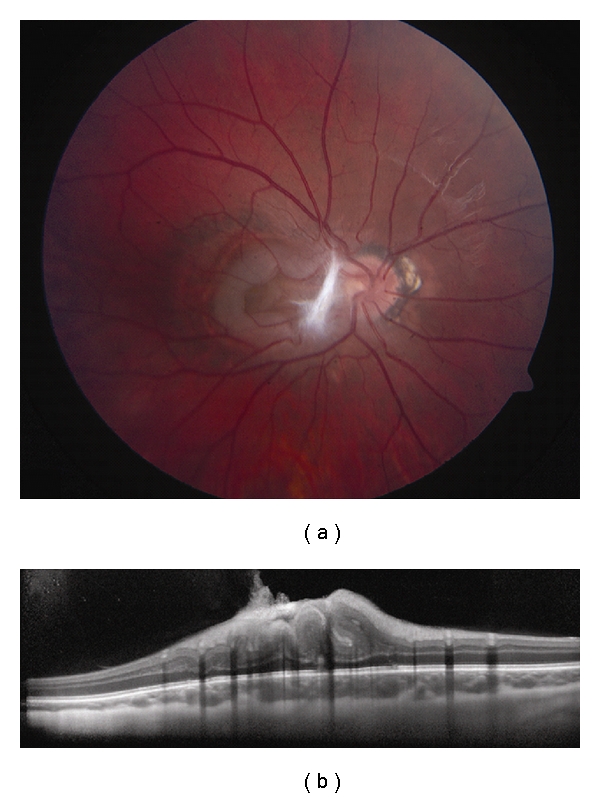
Combined hamartoma of the retina and RPE. (a) Macular combined hamartoma of the retina and RPE with dense preretinal fibrosis at its nasal border. (b) EDI OCT demonstrates gradual transition from the normal adjacent inner retinal layers to a disorganized mass with an overlying tuft of preretinal fibrosis. The outer plexiform layer, external limiting membrane, photoreceptor inner segment-outer segment junction, the RPE, and underlying choroid are intact.

Shields and colleagues described time domain OCT features in 11 cases of combined hamartoma of the retina and RPE, reporting anatomic disorganization with loss of identifiable retinal layers in all cases [46] (Figure 8). OCT evidence of epiretinal membrane was found in 91%, and mean retinal thickness was 766 um [46]. In their series, OCT images showed intact RPE in cases without significant posterior shadowing. Spectral domain OCT has been reported recently but did not add significant information to traditional time domain OCT [47]. 

## 10. Retinoblastoma

Retinoblastoma is the most common primary intraocular tumor in children. What used to be a malignancy with a dismal survival rate now has the highest cure rate in developed countries with the introduction of chemoreduction [48]. Retinoblastoma appears as a yellow-white retinal mass with feeding vessels when entirely intraretinal. When exhibiting an endophytic growth pattern, it is characterized by overlying vitreous seeds. Exophytic retinoblastoma, on the other hand, is associated with serous retinal detachment and occasional subretinal seeds. A rare diffuse pattern of growth is characterized by horizontal rather than vertical growth and can masquerade as uveitis [49]. 

**Figure 9:**
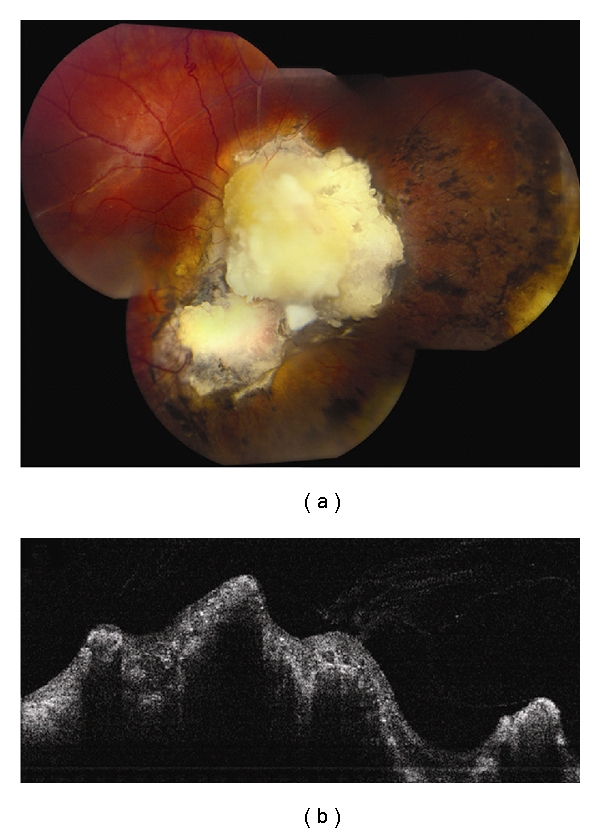
Retinoblastoma. (a) Mostly calcified retinoblastoma following chemoreduction and consolidation. (b) Time domain OCT demonstrates disorganization and irregularity of the inner retinal layers and posterior shadowing from calcification.

Individual retinoblastoma tumors appear on OCT as thickening and disorganization of the neurosensory retina with posterior shadowing possibly from inherent calcification [5] (Figure 9). Associated subretinal fluid or intraretinal cysts are clearly visualized on OCT when present [5, 50]. The role of OCT lies in its ability to image the macula, particularly the fovea. This is important in children with retinoblastoma, since restoration of normal foveal anatomy may be achieved following treatment [51]. Further, differentiation from an organic (i.e., macular edema and loss of photoreceptors) versus a nonorganic (i.e., amblyopia) cause of vision loss is essential to plan for long-term visual rehabilitation and maximizing outcome in all patients.

OCT images of retinoblastoma are difficult to obtain because traditional OCT imaging needs a certain degree of cooperation that is difficult to expect from children. This inherent limitation of OCT machines made reports on OCT of retinoblastoma few and lacking. Today, the development of handheld OCT has revolutionized intraoperative imaging capabilities that could have a tremendous impact on managing childhood ocular diseases such as retinopathy of prematurity and retinoblastoma [52]. These can deliver high-resolution spectral domain OCT images with 3D reconstruction capabilities [52]. 

## 11. Retinal Astrocytic Hamartoma

Retinal astrocytic hamartoma (astrocytoma) is a benign, vascularized, and glial tumor of the retina that may be acquired or congenital. Acquired astrocytic hamartomas appear as yellow-white mass of the inner retina that may be associated with adjacent retinal traction, cystoid macular edema, exudation, and nondilated feeder vessels. They generally lack calcification compared with the congenital form but are otherwise similar ophthalmoscopically. Congenital astrocytic hamartomas present in younger patients, may acquire intrinsic calcification through time, and are sometimes associated with central nervous system astrocytomas in the tuberous sclerosis complex. Both forms often do not require treatment but may at times exhibit progressive growth that leads to blindness and eye pain from neovascular glaucoma that require enucleation [53]. 

**Figure 10:**
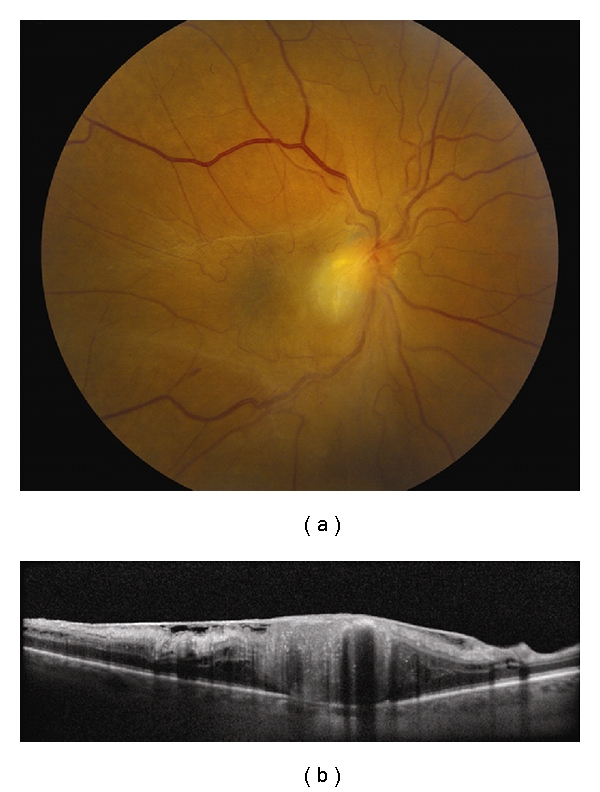
Retinal astrocytic hamartoma. (a) Juxtapapillary retinal astrocytic hamartoma with preretinal fibrosis and nasal dragging of the macula. (b) Spectral domain OCT image exhibits disorganization of the inner retinal layers and an intact RPE underlying the tumor. There is posterior shadowing presumably from calcification of the tumor apex. A dense epiretinal membrane causes the traction of the adjacent normal retina.

Astrocytic hamartomas show inner retinal thickening and disorganization with a gradual transition to the adjacent normal retina [5, 54] (Figure 10). Calcified tumors have higher reflectivity and greater posterior shadowing, while noncalcified tumors allow some light transmission to demonstrate intact outer retinal layers [54, 55]. There may be associated retinal traction in 27%, an intrinsic moth-eaten appearance from intratumoral cysts in 67%, and adjacent retinal or macular edema in 47% [54]. When treatment is initiated for macular edema, OCT may be used to follow resolution of subretinal fluid or release of macular traction [56, 57]. High-resolution spectral domain OCT confirms the intact outer retinal structures, as well as the underlying choriocapillaris [58]. 3D reconstruction demonstrates tumor architecture and its relationship to the adjacent retina in a single image [58]. 

## 12. Retinal Cavernous Hemangioma

Cavernous hemangioma is a benign retinal vascular tumor that appears as dark-red saccular aneurysms. This tumor can be associated with overlying preretinal fibrosis, vitreous hemorrhage, or vascular occlusion [28, 59]. Retinal exudation and edema are typically not associated [28]. There is a familial tendency from mutation of the cerebral cavernous malformation gene located in chromosome 7 in which cerebral or cutaneous cavernous hemangiomas may also be present [8, 28]. 

**Figure 11:**
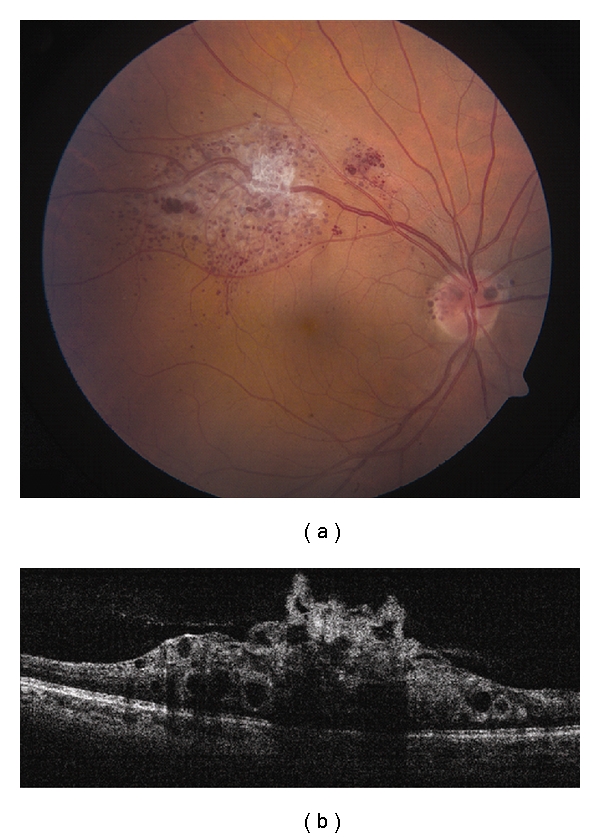
Retinal cavernous hemangioma. (a) Cavernous hemangioma along the superotemporal arcade with associated preretinal fibrosis. Note similar lesions over the optic disc. (b) Time domain OCT shows a lobulated inner retina with optically clear spaces representing the saccular aneurysms. The underlying RPE is intact.

Features of cavernous hemangioma on OCT include lobulated, hyperreflective masses in the inner retina that correspond to the aneurysms. Optically clear cystic spaces may be present within the main hyperreflective mass [8, 60] (Figure 11). A preretinal membrane with traction on the adjacent retina may also be found; subretinal fluid is typically absent. 

## 13. Retinal Hemangioblastoma

Retinal hemangioblastoma (capillary hemangioma) is an orange-red circumscribed vascular lesion with dilated feeding artery and draining vein. When multifocal, bilateral, or occurring in children less than 10 years, they are more often associated with von Hippel-Lindau disease [61]. This tumor can be associated with adjacent retinal exudation or remote cystoid macular edema. Advanced cases may have extensive serous retinal detachment, or neovascular glaucoma [28]. 

**Figure 12:**
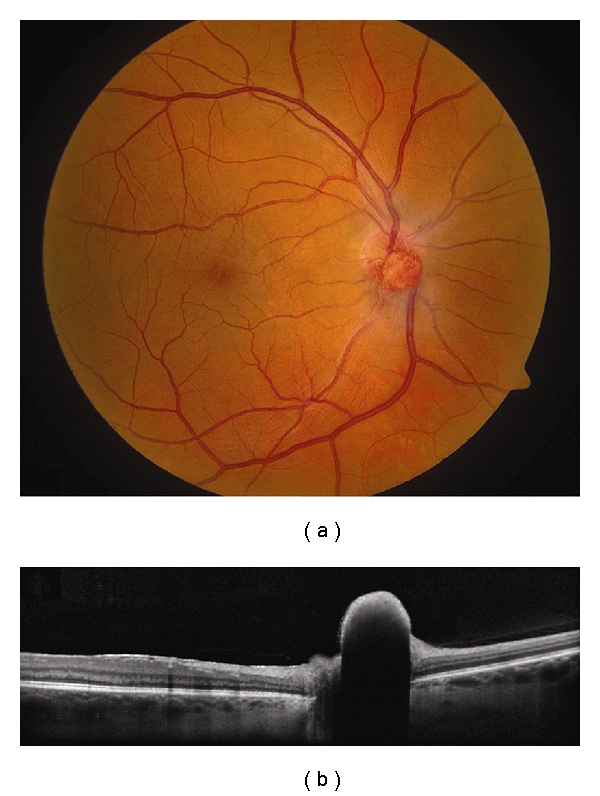
Hemangioblastoma. (a) Optic disc hemangioblastoma with a faint epiretinal membrane along the nasal edge of the fovea. (b) EDI OCT demonstrates a dome-shaped elevation of the inner retina with abrupt transition to the adjacent normal tissue and complete shadowing of the posterior layers. The adjacent RPE and choriocapillaris are intact.

On OCT, retinal hemangioblastoma appears as an optically dense inner retinal mass with posterior shadowing due to intrinsic diffuse capillary channels [5, 8] (Figure 12). OCT is used mostly for the detection of macular edema, epiretinal membrane, and subretinal fluid associated with retinal hemangioblastoma [5, 8]. It is particularly useful for monitoring response to treatment. 

## 14. Retinal Vasoproliferative Tumor

The vasoproliferative tumor of the ocular fundus is usually a unilateral, solitary, and yellow-red retinal lesion located in the inferotemporal periphery with minimally dilated or nondilated feeding artery and draining vein in contrast to hemangioblastoma. Most cases are primary, but 26% are secondary and associated with retinitis pigmentosa, pars planitis, and posterior uveitis, among others [62]. They may have associated macular edema, epiretinal membrane, exudative retinal detachment, or vitreous hemorrhage.

**Figure 13:**
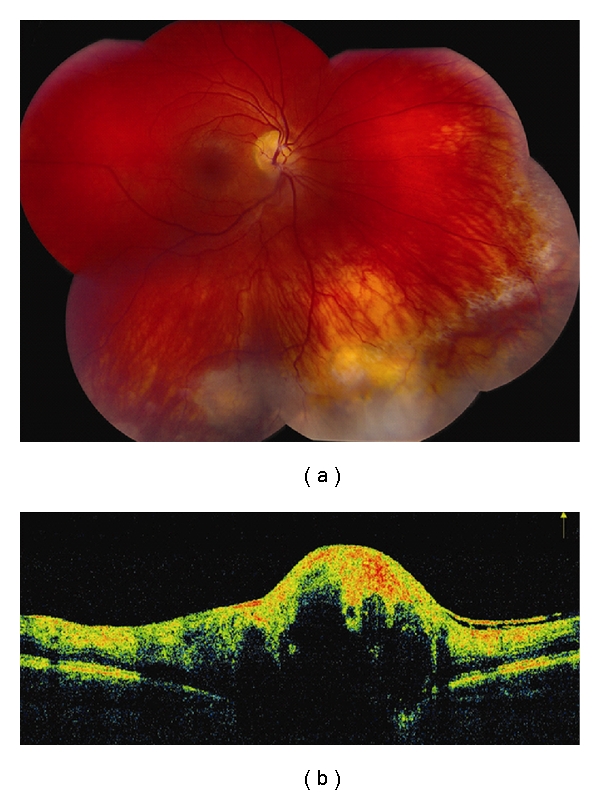
Vasoproliferative tumor. (a) Vasoproliferative tumor located at inferior periphery with preretinal fibrosis at its superior border and yellow subretinal fibrosis nasally. (b) Time domain OCT reveals a hyperreflective and disorganized inner retina with shadowing of the posterior layers including the RPE. An epiretinal membrane is seen at its posterior border.

Inner retinal layer disorganization and posterior shadowing are features of vasoproliferative tumors on OCT [63] (Figure 13). They are difficult to image with OCT due to their peripheral location, but newer machines with longer scan lengths may be useful. OCT is beneficial for detecting associated preretinal fibrosis, macular edema, and subretinal fluid, as well as monitoring treatment [64]. 

## 15. Enhanced Depth Imaging Optical Coherence Tomography

Choroidal visualization has been rendered easier and more precise than before thanks to enhanced depth imaging (EDI) spectral domain OCT. The difficulty faced with conventional spectral domain OCT in imaging the choroid includes decreasing resolution and sensitivity with increasing depth beyond the retina, wavelength-dependent light scattering by RPE and choroid, and the limited 40 decibel dynamic range inherent in Fourier domain systems. In EDI, the instrument is displaced to image deeper layers, and an inverted image is obtained with the superficial layers imaged at the bottom and the deeper layers imaged at the top. This image when flipped is comparable to the conventional spectral domain OCT image but with the choroid and inner sclera visualized at a higher resolution and sensitivity [65]. EDI OCT features have been described in normal choroid, age-related macular degeneration, and myopia [66–68]. This technique can be valuable for studying the structure and extent of choroidal tumors. A small pilot study suggested that small choroidal tumors (<1.0 mm in thickness and <9.0 mm in diameter), that are not detectable by ultrasonography, can be objectively measured by this technique [69]. Notably, even with high-quality choroidal images obtained, the retinal image quality is not compromised. In this preliminary study, EDI OCT showed promise in its ability to measure tumor thickness and visualizing internal structure. Its role in ocular oncology is hopeful, but further studies are still needed to better understand its histopathologic correlation. 

## 16. Swept Source Imaging Optical Coherence Tomography

On the horizon is even higher-grade technology with “swept source” imaging. This employs a long wavelength light source that, at each point, a wavelength of light is rapidly swept across a band of wavelengths with a resultant signal detected by a sensitive photodiode. The photodiode is more sensitive and quicker than the charge-coupled devices (CCDs) used in spectral domain OCT. This extremely fast scan can produce 101,000 A scans per second. Imaging of both the retina and choroid is excellent with good deep penetration into the choroid due to the longer wavelength. 

## 17. Conclusion

Conventional OCT is a valuable tool to visualize anatomic alterations induced by retinal and choroidal tumors. Newer OCT methods with EDI and swept source OCT can allow in vivo cross-sectional imaging of choroidal tumors with details on the tumor structure and measurement of tumor thickness too thin for measurement by ultrasonography.
